# The Role and Regulation of ABI5 (ABA-Insensitive 5) in Plant Development, Abiotic Stress Responses and Phytohormone Crosstalk

**DOI:** 10.3389/fpls.2016.01884

**Published:** 2016-12-16

**Authors:** Anna Skubacz, Agata Daszkowska-Golec, Iwona Szarejko

**Affiliations:** Department of Genetics, Faculty of Biology and Environmental Protection, University of SilesiaKatowice, Poland

**Keywords:** ABI5, abiotic stress response, phytohormone crosstalk, abscisic acid, brassinosteroids, gibberellin acid, jasmonates, posttranslational modification

## Abstract

ABA Insensitive 5 (ABI5) is a basic leucine zipper transcription factor that plays a key role in the regulation of seed germination and early seedling growth in the presence of ABA and abiotic stresses. ABI5 functions in the core ABA signaling, which is composed of PYR/PYL/RCAR receptors, PP2C phosphatases and SnRK2 kinases, through the regulation of the expression of genes that contain the ABSCISIC ACID RESPONSE ELEMENT (ABRE) motif within their promoter region. The regulated targets include stress adaptation genes, e.g., LEA proteins. However, the expression and activation of *ABI5* is not only dependent on the core ABA signaling. Many transcription factors such as ABI3, ABI4, MYB7 and WRKYs play either a positive or a negative role in the regulation of *ABI5* expression. Additionally, the stability and activity of ABI5 are also regulated by other proteins through post-translational modifications such as phosphorylation, ubiquitination, sumoylation and *S*-nitrosylation. Moreover, ABI5 also acts as an ABA and other phytohormone signaling integrator. Components of auxin, cytokinin, gibberellic acid, jasmonate and brassinosteroid signaling and metabolism pathways were shown to take part in *ABI5* regulation and/or to be regulated by ABI5. Monocot orthologs of *AtABI5* have been identified. Although their roles in the molecular and physiological adaptations during abiotic stress have been elucidated, knowledge about their detailed action still remains elusive. Here, we describe the recent advances in understanding the action of ABI5 in early developmental processes and the adaptation of plants to unfavorable environmental conditions. We also focus on ABI5 relation to other phytohormones in the abiotic stress response of plants.

## Introduction

Abiotic stresses, such as drought, salinity or heat, have become a major threat for crop plant survival and yield. Full understanding of the mechanisms ensuring plant adaptation to stress can assist in obtaining tolerant varieties. ABA, which is the major stress phytohormone, takes part in a plant’s adaptation to stress through regulation of physiological processes such as the biosynthesis of osmolytes and the detoxification of ROS (Reactive Oxygen Species); (reviewed by Roychoudhury et al., 2013; Mehrotra et al., 2014; Yoshida et al., 2014; Munemasa et al., 2015; Jones, 2016; Sah et al., 2016). Generally, ABA is considered to be a shoot and root growth inhibitor that acts to save water and energy under stress. However, in some cases ABA promotes root growth and enables the root system to access water in deeper soil layers (Sharp et al., 2004; Skirycz and Inzé, 2010; Zhao et al., 2014). ABA also prevents turgor loss under conditions of reduced water availability. One of the earliest protective processes that are observed in stressed plants is stomatal closure. The endogenous ABA level is precisely controlled by biosynthesis and the catabolism pathways. ABA biosynthesis is a multistage process that is dependent on many enzymes including NCED (CAROTENOID CLEAVAGE DIOXYGENASE). ABA catabolism requires ABA8′hydroxylase activity and results in the creation of phaseic acid (reviewed by Mehrotra et al., 2014; Sah et al., 2016). The perception of an ABA signal is mainly dependent on the core ABA signaling, which includes the action of PYR/PYL/RCAR (PYRABACTIN RESISTANCE PROTEINS/PYR-LIKE PROTEINS/REGULA TORY COMPONENTS OF ABA RECEPTOR) receptors, PPC (PHOSPHATASE 2C) phosphatases and SnRK2 (SNF1-RELA TED PROTEIN KINASE 2) kinases. Finally, AREB/ABF (ABS CISIC ACID RESPONSIVE ELEMENTS-BINDING FACTOR) transcription factors bind to ABRE (ABA RESPONSIVE ELEMENT) elements and regulate the expression of stress responsive genes (reviewed by Nakashima and Yamaguchi-Shinozaki, 2013; Yoshida et al., 2014; Daszkowska-Golec, 2016). ABA-dependent transcription factors are primarily members of large families such as bZIP (BASIC LEUCINE ZIPPER) (Banerjee and Roychoudhury, 2015), MYB (MYELOBLASTOSIS) (Golldack et al., 2011), WRKY (WRKY DNA-BINDING PROTEIN) (Chen L. et al., 2012) or AP2 (APETALA 2) (Golldack et al., 2011). ABI5 (ABA INSENSITIVE 5), which is a bZIP transcription factor, functions in the core ABA signaling.

The effect of mutation in *ABI5* gene was firstly described by Finkelstein’s (1994) group. *Arabidopsis* insertional mutant *abi5* was characterized as ABA insensitive in comparison to the wild type during seed germination. The mutation was mapped on 2 chromosome (Finkelstein, 1994). Transcriptomic analysis of *abi5* mutant indicated the lower level of expression of stress responsive genes. It suggested the role of *ABI5* in abiotic stress response (Finkelstein and Lynch, 2000). Further *ABI5*-related expression analysis confirmed its role in drought and salt adaption during seedling development (Finkelstein and Lynch, 2000; Lopez-Molina et al., 2001; Nakamura et al., 2001). The ABI5-regulated inhibition of seed germination and early seedling growth protects against plant development in adverse conditions. However, the ABI5 function is not only restricted to embryo tissues and its role was also described in the vegetative stage of development (Brocard et al., 2002; Kong et al., 2013). The target genes of ABI5 enable further adaptations to abiotic stresses.

In this review, we present the current understanding of the way ABI5 functions as (1) a regulator of abiotic stress responses and (2) an integrator of ABA crosstalk with other phytohormones. The results that are described primarily refer to research on *Arabidopsis thaliana* as the model plant.

## Seed Germination Under the Control of ABA in the Presence of Abiotic Stress

The process of seed germination is a critical stage in the plant life cycle and therefore plants have evolved precise mechanisms for its regulation (Debeaujon et al., 2000; Manz et al., 2005; Fait et al., 2006; Finch-Savage and Leubner-Metzger, 2006; Nonogaki et al., 2010; Rajjou et al., 2012).

Gibberellic acid (GA) and ABA are the main phytohormones that participate in the regulation of the seed germination process (reviewed by Jacobsen et al., 2002; Daszkowska-Golec, 2011). GA is well known as a positive regulator of seed germination. An environment that is favorable for seed germination leads to the activation of the GA biosynthesis genes – *GA3OX1* (*GIBBERELLIN 3-OXIDASE 1*) and *GA3OX2*, which results in a higher content of the active GA pool (Ogawa et al., 2003; Mitchum et al., 2006). Next, GA responsive genes encoding key regulators and enzymes for germination are activated. The increase of GA during seed germination is associated with a decrease of ABA. The low ABA level is caused by the activity of *CYP707A2* (*CYTOCHROME P450*) encoding ABA8′-hydroxylase and is responsible for ABA catabolism in seeds (Kushiro et al., 2004; Miransari and Smith, 2014). On the contrary, seed dormancy, which prevents germination under unfavorable conditions, is connected with a high ABA and low GA content (Okamoto et al., 2006; Liu et al., 2014). To summarize, the complex ABA and GA interplay enables seed germination at the appropriate moment.

Sometimes, seeds start to germinate prior to the occurrence of abiotic stress. The inhibition of germination and prevention of germinating embryo from dryness is conducted through the ABA-dependent pathway described above. However, plants develop a special subtype of ABA action, which requires the action of seed specific, ABA-dependent transcription factors such as ABI3, ABI4, and ABI5. Mutations in these *ABI* loci lead to an ABA, salt and osmotic stress insensitivity during seed germination (Finkelstein, 1994; Carles et al., 2002; Nambara et al., 2002). ABI3 has a B3 domain and activates genes via an Ry/Sph, seed-specific enhancer element that is present in the promoter sequences. Its function is mainly attributed to the maintenance of embryogenesis and seed dormancy. The loss of this function results in damaged seed development (Finkelstein and Somerville, 1990). ABI3 induces the expression of *MIR159* encoding the negative regulator of *MYB33* and *MYB101.* Both of these act as positive components of ABA signaling. This indicates that ABI3 is responsible for the negative feedback regulation of the ABA cascade in germinating seeds that are under abiotic stress (Reyes and Chua, 2007). ABI4, a transcription factor with an AP2 domain, also functions during seed germination under abiotic stress. The target genes of ABI4 have CE1 (COUPLING ELEMENT 1) in their promoter regions. ABI4 was shown to play a significant role in the ABA-dependent inhibition of lateral root development (De Smet et al., 2003; Shkolnik-Inbar and Bar-Zvi, 2010). Additionally, it has a negative influence on the expression of photosynthesis-related genes and mediates the plant response to glucose (Acevedo-Hernández et al., 2005; Bossi et al., 2009). ABI4 is also assumed to participate in the regulation of ABA signaling through the activation of the *MIR159b* expression and it also targets mature miR159 – *MYB33* and *MYB101* (Daszkowska-Golec et al., 2013). ABI3 and ABI4 were observed to act together with ABI5 in the regulation of abiotic stress responses (Nakamura et al., 2001; Reeves et al., 2011).

## ABI5 – the Crucial Regulator of Seed Germination Process

In germinating seeds and young seedlings, ABA signal perception is also governed by the ABI5 bZIP transcription factor. ABI5 shows a high homology to AREBs and also functions in a similar manner (Finkelstein and Lynch, 2000; Kim, 2006; Yoshida et al., 2010). Its expression is activated by drought and salt stress during seed germination within a short developmental window, which occurs between 48 and 60 h after stratification. Therefore, ABI5 activity causes the inhibition of seed germination or early seedling growth. A correlation was shown between ABA sensitivity, the expression of *ABI5* and the re-establishment of desiccation tolerance (Maia et al., 2014). Growth inhibition of the embryo and root at a later stage of development ensures more water retention and thus drought tolerance (Lopez-Molina et al., 2001; Brocard et al., 2002; Maia et al., 2014). Under stress, SnRK2.2, SnRK2.3 and SnRK2.6 phosphorylate the ABI5 trans-activation domain in vegetative tissues. The phosphorylation of ABI5 changes its conformation and enables its further interactions with other proteins (Nakamura et al., 2001). The first targets of ABI5 that were identified were the genes encoding LEA proteins, *EM1* (*EARLY METHIONINE-LABELED 1*), *EM6* and *LEAD34* (Finkelstein and Lynch, 2000). Additionally, in a Yeast One-Hybrid assay, ABI5 was shown to bind directly with the ABRE sequence in the *EM6* promoter (Nakamura et al., 2001; Carles et al., 2002).

**FIGURE 1:**
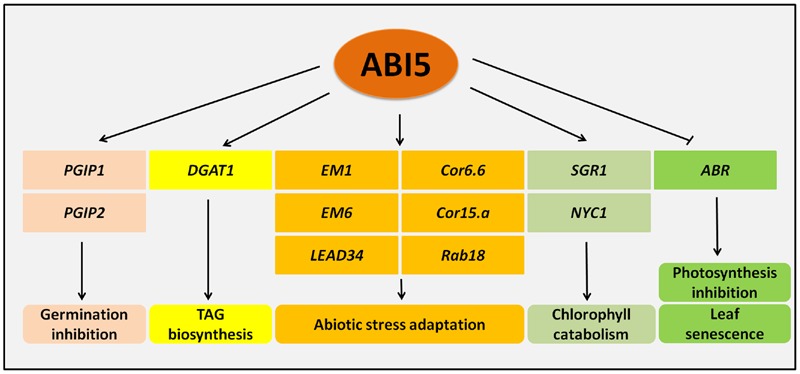
**Genes regulated by ABI5.** ABI5 (ABA INSENSITIVE5) activates the expression of *PGIP* (*POLYGALACTURONASE INHIBITING PROTEIN*) genes encoding polygalacturonase inhibitors, which in turn inhibits seed germination. Another ABI5 target, *DGAT1* (*DIACYLGLYCEROL ACYLTRANSFERASE 1*), participates in TAG (TRIACYLGLYCEROL) biosynthesis and ensures adaptation to stress in seedlings. A group of *LEA* (*LATE EMBRYOGENESIS ABUNDANT*) genes is also positively regulated by ABI5 under abiotic stress. Additionally, ABI5 negatively influences the chlorophyll content, photosynthesis efficiency and leaf senescence via promotion of *SGR1* (*STAYGREEN1*), *NYC1* (*NON-YELLOW COLORING 1*) and repression of *ABR* (*ABA RESPONSIVE PROTEIN*).

Many ABI5 target genes are associated with the germination process, e.g., *PGIP1* (*POLYGALACTURONASE INHIBITING PROTEIN 1*) and *PGIP2* genes (Kanai et al., 2010) (**Figure 1**). The induction of their ABI5-mediated expression leads to the inhibition of the activity of polygalacturanases, which results in the retardation of seed coat rupture and thus germination. In these processes, *ABI5* is under the negative regulation of *PED3* (*PEROXISOME DEFECTIVE 3*), which encodes the ATP-binding cassette transporter that is associated with fatty acid β oxidation. Thus, ABI5 acts as a crucial regulator of germination through its involvement in physiological and the biochemical aspects of this process (Kanai et al., 2010) (**Figure 1**).

## Further Seedling Development is Not Possible Without ABI5 Functioning

Some reports indicate that the ABI5 function is not only restricted to germination and post-germinative growth, but also includes the vegetative tissues. *ABI5* expression has been observed in root tips, nodes and leaf veins at the seedling stage, while in older plants, its activity has been shown at the edges of leaves and in flowers. Additionally, many target genes are regulated by ABI5 at the later stages of plant development. ABA-induced expression of *EM1* was decreased in the vegetative tissues of an *abi5* mutant and *ABI5* overexpression in 2-week-old plants was observed to have some positive impact on the expression of the ABA-responsive genes *Cor6.6, Cor15a* and *Rab18* (Finkelstein and Lynch, 2000; Brocard et al., 2002). In 7-day-old seedlings, ABI5 regulates the expression of *DGAT1* (*DIACYLGLYCEROL ACYLTRANSFERASE 1*) in the presence of salt and osmotic stress in an ABA-dependent way. *DGAT1* encodes a key enzyme in TAG (TRIACYLGLYCEROL) biosynthesis, which is accumulated in stressed plants (Kong et al., 2013). The involvement of ABI5 in the regulation photosynthesis is another example of its function in vegetative tissues. The genes that are responsible for chlorophyll catabolism, *SGR1* (*STAYGREEN1*) and *NYC1* (*NON-YELLOW COLORING 1*), contain an ABRE element in their promoters and are positively regulated by ABI5 together with another transcription factor, EEL (ENHANCED EM LEVEL) (Sakuraba et al., 2014) (**Figure 1**). These results indicate that ABI5 acts as a negative regulator of photosynthesis through the activation of chlorophyll degradation.

Lateral roots development is also regulated by ABI5. Similar to ABI4, ABI5 has an influence on the lateral root length. An *abi5* mutant showed a weakened response to ABA- and nitrate-mediated lateral root growth inhibition (Signora et al., 2001). Additionally, ABA induces *ABI5* expression in the lateral root tips. These results indicate that ABI5 acts as a negative regulator of lateral root development in the presence of stress (Brocard et al., 2002; De Smet et al., 2003; Shkolnik-Inbar and Bar-Zvi, 2010).

ABI5 and ABI4 not only act together during lateral root formation. The ABI5-regulated gene, *DGAT1* encoding the TAG (TRIACYLGLYCEROL) biosynthesis enzyme is also activated by ABI4 (Kong et al., 2013). Furthermore, many genes encoding LEA, dehydrins and oleosins were shown to be regulated by both ABI4 and ABI5. Additionally, ABA pathway-related transcription factors and regulators were identified in the group of genes/proteins that are commonly regulated by ABI4 and ABI5. Among them, negative ABA regulators such as AHG1 (ABA-HYPERSENSITIVE GERMINATION 1) were also detected. Thus, both ABI transcription factors are simultaneously a part of a positive and negative feedback loop in the ABA-signaling. Furthermore, ABI5-specific targets seem to demand other co-regulators compared to ABI4-regulated genes (Reeves et al., 2011).

## The Role of ABI5 in Senescence Process

Recently, ABI5 was shown to take a part in a dark-induced leaf senescence process. It negatively and directly regulates *ABR* (*ABA RESPONSIVE PROTEIN*) expression in the darkness. *ABR*, a gene encoding a LEA protein, is a stress responsive gene that is associated with leaf rescue from the senescence process. The probable mechanism was shown to be related to the photosynthesis proteins. Thus, ABI5 plays a positive role in leaf senescence through its negative impact on the photosynthesis process (Su et al., 2016) (**Figure 1**).

## *ABI5* is Regulated By Multiple Transcription Factors and Other Protein Regulators

**FIGURE 2:**
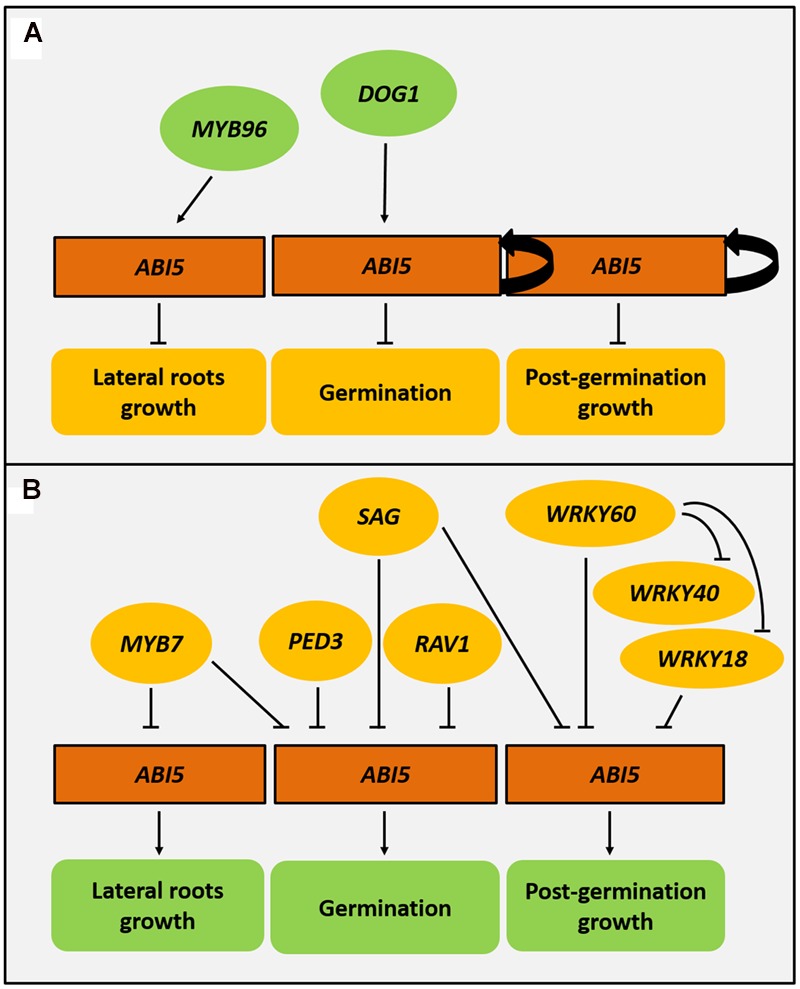
**Regulatory genes that control *ABI5* expression.** *ABI5 (ABA INSENSITIVE5)* is positively regulated by the ABI5 protein thus ensuring a positive feedback loop in ABA signaling. *DOG1* (DELAY OF GERMINATION 1) and *MYB96* also exert a positive effect on ABI5 expression during germination and lateral root formation, respectively. A higher activity of *ABI5* leads to the inhibition of germination, post-germination growth and lateral root formation **(A)**. A group of regulators such as *PED3* (PEROXISOME DEFECTIVE 3), *SAG* (SENSITIVITY TO ABA DURING GERMINATION), *RAV1* (RELATED TO ABI3/VP1), and *MYB7* downregulate *ABI5* expression during seed germination. MYB7 also negatively regulates lateral root formation. *WRKY18* (WRKY DNA-BINDING PROTEIN18), *WRKY40, WRKY60* and SAG repress *ABI5* and enable post-germination growth **(B)**.

Regulation of the *ABI5* expression is complex and is mediated through many regulators. *ABI5* expression is under the control of multiple transcription factors and proteins that belong to other functional groups (**Figure 2**).

### Positive Regulators of ABI5

ABI3 and ABI4 transcription factors are positive regulators of the expression of *ABI5* in the ABA-dependent pathway during both seed germination and early seedling development (Finkelstein and Lynch, 2000; Söderman et al., 2000; Lopez-Molina et al., 2002; Bossi et al., 2009). Furthermore, ABI5 activates its own expression by binding to the *ABI5* promoter (Brocard et al., 2002) (**Figure 2A**). However, in the presence of abiotic stress the negative feedback loop in ABA signaling ensures the balance in ABI5 mediated responses. Some ABA-dependent components, e.g., PYL8 act contrary to general ABA action (Zhao et al., 2014). Stress-activated transcription factors, like MYB7, can inhibit ABI5 expression (Kim et al., 2015). Additionally, ABI5 itself induces expression of *AHT1* (ABA-HYPERSENSITIVE BTB/POZ PROTEIN 1), its negative regulator (Kim et al., 2016). DOG1 (DELAY OF GERMINATION 1) also positively promotes *ABI5* activity. It encodes an unknown protein that is associated with seed dormancy and the repression of the germination-associated genes. It was also shown that DOG1 is another regulator of *ABI5* expression. Possibly, DOG1 acts as a positive regulator of *ABI5* and as a result, activates many *LEA* and *HSP* (*HEAT SHOCK PROTEIN*) genes. Additionally, DOG1 may function together with ABI3 in the regulation of seed maturation (Dekkers et al., 2016) (**Figure 2A**).

MYBs are another large group of transcription factors that are associated with abiotic stress responses (Reyes and Chua, 2007; Dubos et al., 2010). MYB96 is a negative regulator of lateral root formation and participates part in the plant response to drought and salt stress. Since the expression of *ABI5* is activated by MYB96, it supports the role of ABI5 in the ABA-dependent inhibition of lateral root growth (Seo et al., 2009) (**Figure 2A**).

### Negative Regulators of ABI5

Interestingly, the other MYB transcription factor from the R2R3 subgroup, MYB7, also participates in the ABA-mediated regulation of salt and osmotic stress via *ABI5*. MYB7 represses *ABI5* expression during seed germination. Additionally, MYB7 positively influences the content of anthocyanins, which are crucial pigments in the abiotic stress response. In the presence of salt, the *myb7* mutant produces shorter lateral roots. Thus, MYB7 is a positive regulator of lateral root growth under salt stress (Kim et al., 2015) (**Figure 2B**). These studies clearly suggest that the regulation of *ABI5* through MYB96 and MYB7 transcription factors may be essential for ABA-mediated regulation in the context of lateral root development under stress.

In the presence of ABA, SnRK2.2, SnRK2.3 and SnRK2.6 interact and phosphorylate RAV1 (RELATED TO ABI3/VP1), that is a common repressor of ABI3, ABI4, and ABI5. RAV1 activity decreases as the *ABI5* represses the expression of *EM1* and *EM6* encoding LEA proteins (Feng et al., 2014) (**Figure 2B**). Another example is the SAG (SENSITIVITY TO ABA DURING GERMINATION) with a MDN1 (MIDASIN HOMOLOGUE 1) domain. SAG, a negative regulator of ABA signaling, inhibits the expression of *ABI5* and *ABI3* during seed germination and seedling development (Chen et al., 2014) (**Figure 2B**). WRKYs, transcription factors that belong to a large family in plants, also influence the expression of *ABI* loci. They contain the WRKY motif in the N-terminal end and bind to W-box that is present in the regulated promoters. WRKYs are well known to be both positive and negative regulators of the stress response (Chen et al., 2010; Chen L. et al., 2012; Ding et al., 2015). Initially, WRKY40 was described as a negative regulator of ABA signaling through its negative impact on *ABI5* (Shang et al., 2010). Then, WRKY18 and WRKY60 were detected as *ABI5* regulators. WRKY18, WRKY40 and WRKY60 interact with the W-boxes motifs within the promoter regions of *ABI5* and in *ABI4*, and thus repress their expression. The mechanism of this regulation is complex and is restricted to post-germination growth. In the case of *ABI5* regulation, WRKY60 inhibits the binding of WRKY18 and WRKY40 to the *ABI5* promoter, while it simultaneously acts as an *ABI5* repressor. The authors suggest that *ABI5* may be the main target of WRKY action in ABA signaling (Liu et al., 2012) (**Figure 2B**).

AHT1 (ABA-HYPERSENSITIVE BTB/POZ PROTEIN 1), which is a potential substrate receptor of the CRL3 (CULLIN-RING E3 LIGASE3) complex, down-regulates *ABI5*. Interestingly, ABI5 induces *AHT1* in an ABA-dependent manner. Thus ABI5 is a part of the negative feedback regulation of ABA signaling. It can be assumed that this kind of regulation ensures a balanced response of germinating seeds to salt or osmotic stress (Kim et al., 2016).

The regulation of transcript level can be also performed by small RNAs. Interestingly, regulation of *ABI5* expression can be also performed at the post-transcriptional level via miRNA action. In strawberry (*Fragaria* x *ananassa* Duchesne), *FaABI5* is target for Fan-miR73 under control conditions. However, abiotic stresses (salinity, UV-B radiation) downregulate activity of *Fan-miR73* what in turn promotes *FaABI5* and ABA-dependent response. Similar regulation was observed during fruit ripening (Li et al., 2016).

## Epigenetic Regulation of the *ABI5* Gene

*ABI5* also undergoes epigenetic regulation. Together with *ABI3, ABI5* is under the control of PKL (PICKLE), a SWI/SNF class chromatin-remodeling factor. PKL negatively regulates the expression of *ABI5* and *ABI3* because of the increase in the histone methylation (H3K9 and H3K27) of their promoters and chromatin repression in an ABA-dependent manner. PKL releases the germinating embryos from this inhibition and enables seedling growth under unfavorable conditions (Perruc et al., 2007). Another example of *ABI5* epigenetic regulation is the ABA-activated action of HLS1 (HOOKLESS 1), a putative histone acetyltransferase. HLS1 directly interacts with the *ABI5* sequence, mediates H3 acetylation and positively regulates *ABI5* expression. Furthermore, HLS1 acts cooperatively with MED18 (MEDIATOR 18), a subunit of MEDIATOR complex, to increase *ABI5* expression. HLS1 and MED18 interact physically and probably are the part of the same complex. Using transgenic *Arabidopsis* plants it was proved that HLS1 and MED18 are engaged in ABA signaling and are tightly related to ABI5 action (Liao et al., 2016).

An increased activity of TE (Transposable Elements) was observed under unfavorable environmental conditions (McCue et al., 2012; Le et al., 2014; Makarevitch et al., 2015). Recently, the mechanism of the modulation of *ABI5* expression via TE has been described. Heat stress-activated retrotransposon, ONESEN, was integrated into *ABI5* and disrupted its expression, possibly through the incorrect progress of the transcription (Ito et al., 2016). Moreover, the ABA insensitivity, which was mediated through the insertion of ONESEN in the *ABI5* gene, was heritable. On the other hand, an ABA insensitive phenotype can be recovered by the IBM2 (INCREASE IN BONSAI METHYLATION 2) mediated epigenetic regulation of ONESEN. It is possible that ONESEN has an influence on ABA-related genes thus ensuring not only a short-term response to abiotic stress, but also an evolutionary plant adaptation to adverse environmental conditions (Ito et al., 2016).

## ABI5 Regulation at the Protein Level

ABI5 activity is regulated at the protein level via protein interaction and posttranslational modification (reviewed in Daszkowska-Golec, 2016; **Table 1**).

**Table 1:** Posttranslational modifications of ABI5.

Posttranslational modification	Regulators	Result of posttranslational modification	Reference
Phosphorylation	SnRK2s CPK11 PKS5	Activation of ABI5 action	Nakashima et al., 2009; Lynch et al., 2012; Zhou et al., 2015
Dephosporylation	AHG1, AHG3 PP2A FyPP1, FyPP3	Deactivation of ABI5 action	Lynch et al., 2012; Hu et al., 2014; Dai et al., 2013
Ubiquitination	KEG DWA1, DWA2 (CUL4-based patway)	ABI5 proteasomal degradation	Stone et al., 2006; Lee et al., 2010
Sumoylation	SIZ1	Deactivation via ABI5 location in nuclear bodies	Miura et al., 2009
*S*-nitrosylation		ABI5 degradation mediated by KEG and CUL4 ligases	Albertos et al., 2015

### Turning on ABI5 into Action

ABA-related phosphorylation that is mediated by SnRK2 kinases is required for ABI5 stability and activation as a transcription factor (Nakashima et al., 2009). Phosphorylation of ABI5 occurs in three conserved domains that are localized in the N-terminal end of the protein (Lopez-Molina et al., 2002). Three amino acids, Ser-42, Ser-145, and Thr-201, are considered to be the targets for the SnRK2-mediated phosphorylation of ABI5 (Wang Y. et al., 2013).

The stability of the ABI5 protein is regulated through its interaction with other proteins. ABI3 is well known as an interaction partner and enhancer of ABI5 activity at the protein level (Nakamura et al., 2001). It was shown that in the vegetative tissues, ABI5 was able to induce *EM1* and *EM6* expression only when accompanied by the activation domain from VP16 (VIRION PROTEIN 16) protein. These reports suggest that ABI5 acts in concert with another regulator in seedlings, such as ABI3 (Bensmihen et al., 2004). ABI5 is not only self-regulated during transcription. As a bZIP transcription factor, ABI5 is able to create homo- or heterodimers. The ABA-dependent autoregulation of ABI5 was observed at the protein level through the possibility of creating homodimers (Nakamura et al., 2001; Lynch et al., 2012). ABI5 and AREB/ABF transcription factors are considered to have a redundant function and similar binding properties. ABI5 was shown to interact with ABF3 (ABA RESPONSIVE ELEMENTS-BINDING FACTOR 3) and ABF1 (ABA RESPONSIVE ELEMENTS-BINDING FACTOR 1). Heterodimers composed of ABI5 and other AREB/ABFs regulate the expression of stress-responsive genes. Additionally, there is a negative reciprocal regulation between *ABI5* and *ABF3* (Kim et al., 2002; Finkelstein et al., 2005). ABI5 also interacts with other regulators of the ABA pathway such as SnRK2.2, SnRK2.3, AHG1 (ABA-HYPERSENSITIVE GERMINATION 1) and AHG3. This indicates that the core ABA-signaling components regulate the early stages of plant development through the phosphorylation and dephosphorylation of ABI5 (Lynch et al., 2012) (**Table 1**). Additionally, other kinases and phosphatases that regulate the ABI5 phosphorylation status were also identified. Ca^2+^ signaling-related kinases – CPK11 (CALCIUM-DEPENDENT PROTEIN KINASE 11) (Lynch et al., 2012) and PKS5, a member of CIPK/PKSs (CALCINEURIN B-LIKE PROTEIN-INTERACTING PROTEIN KINASEs/PROTEIN KINASEs SOS2-LIKE) (Zhou et al., 2015), mediate ABI5 phosphorylation (**Table 1**). ABI5 dephosphorylation events may be dependent on PP2A (PROTEIN PHOSPHATASE 2A) (Hu et al., 2014) and two catalytic subunits of PP6 (PROTEIN PHOSPHATASE 6), FyPP1 and FyPP3 (Dai et al., 2013) (**Table 1**). However, the interaction between ABI5 and TAP46 protects active and stable ABI5 from the removal of its phosphate groups by PP2A (PROTEIN PHOSPHATASE 2A). It was shown that TAP46 also binds to PP2A, which can prevent the formation of an ABI5-PP2A complex (Hu et al., 2014).

### Turning Off ABI5 via Degradation Processes

ABI5 is dephosphorylated when there is no abiotic stress. ABI5 that has no phosphorylation groups is neither active nor stable and undergoes degradation that is mediated by the 26S proteasome pathway (Lopez-Molina et al., 2001). Ubiquitination and 26S proteasome-mediated protein degradation are processes that are directed by three enzymes – ubiquitin protein ligase (E3), ubiquitin-conjugating (E2) and ubiquitin activating (E1) enzymes. RING E3 ligase, KEG (KEEP ON GOING), interacts with ABI5 and controls its accumulation (Stone et al., 2006) (**Table 1**). ABA counteracts KEG activity and leads to KEG autoubiquitination (Liu and Stone, 2010). The phosphorylation status of ABI5 probably does not influence the interaction between ABI5 and KEG in the absence of ABA (Liu and Stone, 2014). The AFP (ABI FIVE BINDING PROTEIN) family also participates in the control of ABI5 accumulation. Although AFP1 and AFP2 mediate the proteasomal degradation of ABI5, their activity is strictly associated with a precise developmental stage (Garcia et al., 2008). Additionally, ABI5 ubiquitination and degradation can be mediated by CUL4-based (CULLIN 4) E3 ligases. Two proteins, DWA1 (DWD HYPERSENSITIVE TO ABA 1) and DWA2, are involved in this regulation through the binding and marking of ABI5 for degradation (Lee et al., 2010) (**Table 1**).

The abundance ABI5 is not only controlled by ubiquitination. Sumoylation is the process of the attachment of the SUMO (SMALL UBIQUITIN-RELATED MODIFIER) group to a protein substrate and is regulated by an E1-activating enzyme, E2-conjugating enzyme and E3 ligase (Geiss-Friedlander and Melchior, 2007). SIZ1, a SUMO E3 ligase, negatively regulates ABI5 activity by targeting its lysine K391. However, ABI5 sumoylation prevents its degradation through the ABI5 location in alternative nuclear bodies. This ensures a pool of inactive ABI5 that is not susceptible to degradation (Miura et al., 2009) (**Table 1**). CRWN1 (CROWDED NUCLEI 1) and CRWN3 proteins were also identified as negative regulators of ABI5 accumulation during seed germination. CRWN3 co-localizes with ABI5 in the nuclear bodies, where it probably mediates its degradation (Zhao et al., 2015).

Recently, ABI5 was shown to be *S*-nitrosylated at cysteine 153. This type of modification leads to the degradation of ABI5 mediated by the CUL4 (CULLIN4) and KEG (KEEP ON GOING) E3 ligases and finally ensures seed germination (Albertos et al., 2015) (**Table 1**). It should also be stressed that NO also acts as a negative regulator of ABA signaling during stomatal closure (Wang et al., 2015).

To summarize, ABI5 activity and stability is regulated by multiple posttranslational modifications through the actions of many regulators and enzymes.

## ABI5 as the Integrator of ABA and Other Phytohormone Signaling During Abiotic Stress

Although ABA is considered to be the main stress plant hormone, other phytohormones such as auxin, cytokinins (CKs), gibberellic acid (GA), brassinosteroids (BRs) and jasmonic acid (JA) also regulate plant adaptation to an adverse environment. Many of the components that are responsible for the biosynthesis and signaling of auxin, CKs, GA, BRs, and JA were identified as taking part in the plant response to drought and salt stress. The precise action of the regulators that belong to different phytohormone pathways ensures a balanced reaction of a plant to abiotic stress. The auxin, CKs, GA, BRs and JA regulation of drought and salt stress includes crosstalk with ABA. Many ABA-related genes are under the control of other phytohormones. This usually requires an interaction with the ABA signaling components. Among the many ABA-related regulators, ABI5, which integrates various phytohormone pathways and thus enables appropriate plant stress response, appears to be one of the most important (**Table 2**).

**Table 2:** ABI5 function in crosstalk with other phytohormones.

Phytohormone pathway	Regulator	Function	Type of interaction with the *ABI5* gene or ABI5 protein	Reference
Auxin	PIN1	Auxin transporter	ABI5 negatively regulates PIN1 accumulation in roots	Yuan et al., 2014
Cytokinin	ARR4, ARR5, ARR6 AHK4, AHP2, AHP3, AHP5, ARR12		Down-regulation of *ABI5* expression Proteasomal degradation of ABI5 protein	Wang et al., 2011 Guan et al., 2014
Gibberellic Acid	RGL2 RGA	DELLA protein DELLA protein	Reciprocal positive regulation of *ABI5* and *RGL2* expression ABI5 and RGL2 together regulate the expression of *MFT* and *GASA6* ABI5 and RGA together regulate the expression of *MFT* and *SOM*	Piskurewicz et al., 2008; Yuan et al., 2011 Xi et al., 2010; Zhong et al., 2015 Xi et al., 2010; Lim et al., 2013
Jasmonic Acid	MED25 WRKY57	Subunit of MEDIATOR complex Transcription factor	MED25 enhances MYC2 activity and competes with ABI5 to bind ABA-responsive gene promoters. Additionally, MED25 promotes ABI5 degradation. WKRY57 negatively regulates JA signaling and interacts with the *ABI5* promoter	Chen R. et al., 2012 Jiang et al., 2014; Su et al., 2016
Brassinosteroids	BIN2 BZR1	Kinase Transcription factor	BIN2 phosphorylates ABI5 BZR1 negatively regulates the expression of ABI5	Hu and Yu, 2014; Yang et al., 2016

### Role of ABI5 and ABA Signaling in Auxin Pathway Regulation

Auxin positively regulates plant growth and development via the control of cell division, elongation and differentiation. The role of auxin in abiotic stress adaptation is evident, although the precise mechanism of its action still remains unknown. It was shown that a water deficit negatively influences the auxin content (Du et al., 2013; Bao et al., 2014). The biosynthesis of auxin depends on *TAA1* (*L-TRYPTOPHAN PYRUVATE AMINOTRANSFERASE 1*) and the *YUC* (*YUCCA*) gene family (Won et al., 2011). Expression of genes from the *YUC* family is negatively regulated by drought (Du et al., 2013). However, a higher amount of endogenous auxin ensures a better tolerance to water deprivation (Shi et al., 2014).

The existence of an interaction between auxin and the ABA pathways is evident. Many processes that are significant for the regulation of abiotic stress responses such as stomata closure (Tanaka et al., 2006) and lateral root formation (Shkolnik-Inbar and Bar-Zvi, 2010; Zhao et al., 2014) are under the control of ABA and auxin The link between ABI5 and auxins was established when *PIN1* (*PIN-FORMED 1*) action was studied.

*PIN1*, a gene encoding the auxin transporter, showed a decreased expression when osmotic stress was applied (Rowe et al., 2016). Salt stress disturbed the expression of the *PIN* genes and stabilized AXR3/IAA17, which in turn reduced the size of the root meristem because of the lower auxin level (Liu et al., 2015). Yuan et al. (2014) showed that ABI5 participated in the reduction of the root meristem size through a negative impact on the PIN1 content. A glucose-dependent restriction of the root apical meristem was related to a low auxin level and ABI5-mediated regulation. An enhanced expression of *ABI5* resulted in a reduced accumulation of the PIN1 protein, which in turn decreased the number and lengths of root apical meristem cells (Yuan et al., 2014) (**Table 2**). Therefore, ABI5 can be considered to be part of the interaction between auxin and ABA.

### Negative Regulation of ABI5 and ABA Signaling by Cytokinins

Cytokinins belong to another group of plant hormones that regulate the growth and development of plants, mainly through the induction of cell divisions. CK balance is ensured by IPTs (ISOPENTENYLTRANSFERASEs) and CKXs (CK DEHYDROGENASEs), which are the enzymes that are responsible for the biosynthesis and catabolism of CKs, respectively. CKs generally have a negative impact on the adaptation to abiotic stresses. Application of drought or salt stress reduces the amount of CK in plants (reviewed by Ha et al., 2012; Zwack and Rashotte, 2015). ABA and CKs generally act in an opposite manner under stress conditions (Zwack and Rashotte, 2015). ABA negatively regulates the CK biosynthesis genes such as *IPT3* (*ISOPENTENYLTRANSFERASE3*) and *IPT8* and ensures a low CK level under stress conditions (Wang et al., 2011). Interestingly, a CK biosynthesis mutant, *ipt1/ipt3/ipt5/ipt7*, had a decreased endogenous ABA level and showed an increased ABA sensitivity at the same time (Nishiyama et al., 2011). Possibly, there is a strong link between the ABA and CK pathways, whose disruption causes changes in the phytohormonal balance and other types of plant reactions under stress.

ARRs (ARABIDOPSIS RESPONSE REGULATORs) participate in the interaction between ABA and cytokinin under stress where the ABI5 function was highlighted. A triple mutant in the genes encoding the B-type of ARRs, *arr1/arr10/arr12* showed an ABA-hypersensitive reaction. Additionally, it was described as drought tolerant, because of the smaller stomatal aperture, a higher anthocyanin biosynthesis and better cell membrane integrity (Nguyen et al., 2016). The A-type negative regulators, ARR4, ARR5 and ARR6, were shown to down-regulate *ABI5* expression during seed germination. Furthermore, they have the ability to bind ABI5 at the protein level. This indicates that CKs exert a negative effect on ABI5 and ABA signaling integrally. On the other hand, ABA inhibits the expression of *ARR4, ARR5* and *ARR6*. Thus, ABI5 activity is regulated by ABA and CK through ARR4, ARR5 and ARR6 at the same time (Wang et al., 2011) (**Table 2**). CKs also influence ABI5 function during seedling development. However, it includes another part of CK signaling and acts at a different regulatory level compared to seed germination. CKs counteract the arrest of ABA seedling growth via the negative regulation of ABI5 at the protein level. It was shown that the action of the CK signaling components, AHK4, AHP2, AHP3, AHP5 and the B-type regulator, ARR12, promote ABI5 26S proteasomal degradation and stimulate the greening of cotyledons. In this way, CKs enable seedling growth under unfavorable conditions (Guan et al., 2014) (**Table 2**). Therefore, ABI5 is an important component of ABA and CK crosstalk during seed germination and seedling development.

### ABI5 Interactions With Negative GA Regulators during Seed Germination under Abiotic Stress

Gibberellic acid (GA) positively regulates the growth and development of plants primarily during seed germination and the conversion between the vegetative and generative stages. Biosynthesis of GA is dependent on GA3ox (GA 3-OXIDASE) and GA20ox (Hisamatsu et al., 2005; reviewed by Colebrook et al., 2014) activity, while GA catabolism is mediated by GA2ox (Rieu et al., 2008). The GA level is modulated by abiotic stress. In maize (*Zea mays*), the GA content was reduced in its response to drought (Wang et al., 2008). In *Arabidopsis, GA2ox6* and *GA2ox7* expression was up-regulated by osmotic and salt stress, respectively (Magome et al., 2008; Dubois et al., 2013). The GA signal is perceived by the GID1 (GA INSENSITIVE DWARF 1) receptor, which in turn deactivates the DELLA proteins, which are negative regulators of GA signaling (Griffiths et al., 2006). The regulation of GA signaling under abiotic stress through the action of DELLAs has been described in detail (Achard et al., 2008; Claeys et al., 2012).

ABA acts opposite to GA during abiotic stress conditions and DELLAs play a crucial role in this interaction. DELLA proteins are activated when the GA level in cells is low (Tyler et al., 2004; Sun, 2011). They generally exert a positive influence on ABA signaling. The RGL2 (RGA-LIKE 2) protein with a DELLA domain positively influences ABA biosynthesis, probably through a XERICO protein, which in turn stimulates *ABI5* expression during seed germination under conditions of a low GA content. Thus, ABI5 activity can be observed in the presence of a high ABA:GA ratio (Piskurewicz et al., 2008) (**Table 2**). Similar to *ABI5, RGL2* is also up-regulated by salt in seeds. Furthermore, RGL2 and ABI5 exert a positive effect on each other at the level of gene expression and together regulate seed germination and early seedling growth in the presence of salt (Yuan et al., 2011) (**Table 2**). Recently, the role of NF-YC (NUCLEAR FACTORY C) proteins was shown in RGL2-dependent *ABI5* expression activation. NF-YC3, NF-YC4 and NF-YC9 are able to form complex with RGL2 and bind to CCAAT elements present in *ABI5* promoter. As the result, the transcription of *ABI5* is promoted and seed germination is inhibited (Liu et al., 2016). ABI5 and DELLA proteins also regulate a set of the same target genes. *MFT* (*MOTHER OF FT AND TFL 1*) encoding a phosphatidylethanolamine-binding protein is expressed in the radical-hypocotyl transition zone and releases seed germination from an inhibitory ABA effect. The activity of *MFT* is up-regulated during seed germination by ABI5, RGL2 and another DELLA protein, RGA (REPRESSOR OF GA). Additionally, MFT down-regulates the expression of *ABI5*, thus providing the negative feedback loop in ABA signaling if the ABA content is too high during seed germination. *MFT* regulation by the ABI5 and DELLA proteins is part of the mechanism that ensures an appropriate seed germination potential according to the environmental conditions (Xi et al., 2010) (**Table 2**).

Another common ABI5 and RGA target gene, *SOM* (*SOMNUS*), regulates germination under heat stress. *SOM* expression is activated by ABA and inactivated by GA with the participation of ABI5 and RGA. They bind each other at specific motifs within the promoter region of *SOM* and activate its expression in the presence of a high temperature. The action of another DELLA protein, GAI (GA INSENSITIVE), is also possible in the regulation of *SOM* expression. The activity of SOM, a zinc finger protein, represses seed germination through the simultaneous promotion of ABA biosynthesis and the inhibition of GA biosynthesis. In this way, SOM can create a positive feedback loop with *ABI5* and *RGA* (Lim et al., 2013) (**Table 2**). ABI5 and the DELLA protein, RGL2, also regulate seed germination via the ABA-dependent inhibition of *GASA6* (*GIBBERELLIC ACID-STIMULATED ARABIDOPSIS 6*) expression. GASA6 acts as a positive regulator of seed germination under abiotic stress. Under a high GA level, GASA6 activates *EXPA1* (*EXPANSIN A1*) encoding cell wall loosening expansin, which in turn stimulates the elongation of the embryonic axis and seed germination. Regulation of *GASA6* indicates a direct link between ABI5, RGL2 and the inhibition of seed germination (Zhong et al., 2015) (**Table 2**). Thus, ABI5 plays the role of GA-signaling modulator, which leads to the repression of seed germination under unfavorable conditions.

### Modulation of ABI5 and JA Signaling by Common Protein Regulators

Jasmonic acid plays a role that is similar to ABA during abiotic stress and ensures plant adaptation to limited water conditions (Riemann et al., 2015). JA positively regulates stress-adaptive processes such as stomatal closure and the activity of antioxidant enzymes (reviewed by Suhita et al., 2003; Qiu et al., 2014). JA signaling is mediated through JAZ (JASMONATE-ZIM-DOMAIN PROTEIN) repressors and MYC (MYELOCYTOMATOSIS) transcription factors. In the presence of JA, MYCs are released from the JAZ-MYC complexes and activate the expression of JA-responsive genes (Chini et al., 2007; Dombrecht et al., 2007). ABA and JA signaling show a strong link. During stomata closure, the increase of ROS and NO content is the result of the ABA and JA action (Munemasa et al., 2007). It was recently shown that the ABA receptor, PYR6, interacts with MYC2. This suggests a close interaction between the ABA and JA pathways (Aleman et al., 2016). ABI5 also seems to participate in the ABA-JA crosstalk. The activity of ABI5 and MYC2 is modulated at the protein level via the same subunit of the MEDIATOR complex – MED25. The expression of ABA-related genes such as *EM1, EM2* and *RAB18* and JA-responsive genes including *VSP1* (*VEGETATIVE STORAGE PROTEIN 1*), *LOX2* (*LIPOXYGENASE 2*), *JAZ6, JAZ8* is regulated in an opposite manner by MED25. In JA signaling, MED25 acts as a positive regulator through its interaction with MYC2 in the promoter region of the JA-responsive genes. On the other hand, the presence of MED25 in the promoter regions of ABA-responsive genes represses their expression through the prevention of ABI5 binding to the ABRE elements. Additionally, MED25 enhances ABI5 protein degradation (Chen R. et al., 2012) (**Table 2**). Thus, ABI5 and the component of JA signaling, MYC2, are under control of a common regulator, MED25. JA signaling can also modify the expression of *ABI5* through WRKY57 activity. The WRKY57 transcription factor acts as a negative regulator of JA-induced leaf senescence. The activity of JAZ proteins leads to the degradation of WRKY57 (Jiang et al., 2014). Recently, it was shown that WRKY57 interacts with the *ABI5* promoter. This suggests that WRKY57 influences the *ABI5* expression (Su et al., 2016) (**Table 2**). Possibly, WRKY57 can link JA and ABA signaling via *ABI5* regulation.

### ABI5 As a Target of Negative Regulators of BR Signaling

Brassinosteroids are steroid hormones that regulate a wide range of physiological processes in plant life cycle. Two last decades have witnessed a significant advance in the deciphering the molecular mechanisms underlying BR signaling from perception to regulation of transcription factors influencing expression of target genes. It has been reported that some of the components of BR signaling pathway act as multifunctional proteins involved in other signaling networks, such as the signaling cascades of other hormones, regulating diverse physiological processes (reviewed in Gruszka, 2013).

ABI5 was shown to be a target of important brassinosteroid signaling components such as BIN2 (Brassinosteroid-Insensitive2) and BZR1 (Brassinazole-Resistant1). BIN2 is a kinase functioning as a major negative regulator of BR signaling through BZR1 and BES1 inactivation. The BZR1 and BES1/BZR2 (BRI1-EMS-Supressor1/Brassinazole-Resistant2) transcription factors are key elements mediating BR-regulated gene expression in *A. thaliana*. It was reported that these factors bind to promoters of numerous genes involved in signaling and synthesis pathways of GAs, ABA, ethylene, cytokinins and jasmonate, suggesting that BR signaling impacts metabolism of several other plant hormones (Sun et al., 2010; Yu et al., 2011).

It was proved that BIN2 plays important role during seed germination and also in abiotic stress response in ABA-mediated pathway (Yang et al., 2012). BIN2 phosphorylates and stabilizes ABI5 in order to mediate ABA response during seed germination. Contrary, the exogenously applied BRs repress the BIN2-ABI5 interaction and thus antagonize ABA-mediated inhibition of seed germination (Hu and Yu, 2014). Interestingly, it was also shown that BIN2 can interact and phosphorylate Snf-1-related kinase 2s (SnRK2s), SnRK2.2 and SnRK2.3, positive regulators of ABA signaling that act upstream the ABI5 (Cai et al., 2014).

Yang et al. (2016) showed that *bzr1-1D*, a dominant mutant with enhanced BR signaling, was less sensitive to ABA-inhibited primary root growth. ABA INSENSITIVE5 (ABI5) was found to be repressed not only by exogenously applied BR but also by BZR1 itself. BZR1 could bind strongly with several G-box *cis*-elements in the promoter of ABI5 suppressing its expression and further resulting in insensitivity to ABA. Taking these results together it was demonstrated that ABI5 is a direct target gene of BZR1, and modulating the expression of ABI5 by BZR1 plays important roles in regulating the crosstalk between the BR and ABA signaling pathways.

## Function of ABI5 Orthologs in Dicot and Monocot Plants During Abiotic Stress

Arabidopsis ABI5 orthologs have been identified in other dicot plants and their sequences are available in bioinformatic databases. However, their precise function under abiotic stresses was described only for a few species. In *Brassica oleracea, BolABI5* was found as a close homolog of *At*ABI5. *BolABI5* is expressed mainly in flowers. *BolABI5* overexpression in *abi5* rescued ABA insensitive phenotype during seed germination. Additionally, application of ABA, drought, salt and osmotic stress activated *BolABI5* expression and indicated its role in abiotic stress adaptation. Similar to *AtABI5, BolABI5* activates expression of target genes through binding to ABRE elements present in their promoters (Zhou et al., 2013). BolABI5 possesses the ability to interact with BolOST1, a homolog of SnRK2.6/OST1. Probably, BolOST1 is responsible for its phosphorylation and activation as a transcription factor (Wang M. et al., 2013). Two *AtABI5* orthologs, *BrABI5a* and *BrABI5b*, were identified in *Brassica rapa*. Both are able to reverse *abi5* phenotype in the presence of ABA during seed germination. BrABI5a and BrABI5b show a high similarity to ABI5 including conservation of phosphorylation sites. Expression of *BrABI5* genes is activated by ABA treatment; however they act differently in the presence of abiotic stresses. Only *BrABI5b* is induced by drought and salt. Probably they play a different role in the adaption to unfavorable conditions (Bai et al., 2016).

The role of *ABI5* homologs in other dicots was also reported. The expression of *SlABI5* in tomato (*Solanum lycopersicum*) increased in seeds in reponse to ABA (Sun et al., 2015). The function of *Medicaco truncatula AtABI5* homolog, *MtABI5*, was described in more detail, using an insertional *mtabi5* mutant. Similarly to *atabi5, mtabi5* was ABA-insensitive during seed germination. Furthermore, the level of seed dormancy was decreased in *mtabi5*. Expression analysis showed reduced expression of *LEA*s, and *SIP1* (*SEED IMBIBITION1*) encoding raffinose synthase in *mtabi5* seeds, while photosynthesis-related genes, e.g., *LHCA1* (*PHOTOSYSTEM I LIGHT HARVESTING COMPLEX GENE 1*) and *PsaD-2* (*PHOTOSYSTEM I SUBUNIT D-2*) were up-regulated. MtABI5 acts as the seed development regulator through the control of RFO (RAFFINOSE FAMILY OLIGOSACCHARYDES) and LEA synthesis. Additionally, it serves as a repressor of photosynthesis and accumulation of chlorophyll and carotenoids (Zinsmeister et al., 2016). Recently, action of *FaABI5* in strawberry (*Fragaria* x *ananassa* Duchesne) has been also described. It regulates fruit ripening and responses to such as salinity and UV-B radiation (Li et al., 2016). The above results confirm the conserved role of ABI5 orthologs in dicot species. However, further studies are needed in order to reveal their accurate function.

Nowadays, the abiotic stress tolerance of monocot plants is a very important issue. An accurate understanding of the stress-related mechanisms in monocots can help to improve cereal growth and yield in adverse environments. One of the areas of interest is the ABA-dependent regulation of the stress response in cereals, which includes the action of ABI5.

Although AtABI5 orthologs have been identified in monocot plants, their precise function still remains elusive. HvABI5 is a barley bZIP transcription factor that has high similarity to AtAREB2 and AtABI5 (Casaretto and Ho, 2003). The potential phosphorylation sites of HvABI5 are strongly conserved. The role of barley *HvABI5* in abiotic stress responses is very poorly understood. It is already known that HvABI5 directly, in ABA-dependent way, activates the *HVA1* and *HVA22* expression through binding to the ABRC (ABA RESPONSE PROMOTER COMPLEX) elements within their promoters. However, HvVP1 (VIVIPAROUS1), a homolog of AtABI3, is also required for the activation *of HVA1* and *HVA22* expression. *HVA1* and *HVA22* encode, respectively, a group 3 LEA protein and a protein that is involved in vesicular trafficking. Their activity was shown to ensure tolerance to low water availability during seed germination (Casaretto and Ho, 2003). Induction of *HvABI5* expression has also been detected in leaves after drought treatment in different barley varieties (de Mezer et al., 2014). In rice, OsABI5 shows a high homology to ABI5 and HvABI5 and had the ability to bind the G-box element (Zou et al., 2008). Like ABI5, OsABI5 interacts with the AtABI3 ortholog OsVP1. Overexpression of *OsABI5* in an *abi5* Arabidopsis mutant rescued ABA-insensitive phenotype. This could be proof that *AtABI5* and *OsABI5* share similar functions (Zou et al., 2007). *OsABI5* expression is induced by ABA and salt, but drought and cold stress represses its activity. Forty-five-day-old plants of *OsABI5*-overexpression lines showed a faster turgor loss, chlorosis and growth inhibition in the presence of salt. Conversely, *OsABI5*-antisense plants were described as being salt tolerant with a changed expression of *SalT*, a salt-responsive gene and *SKC1*, which is a QTL (Quantitative Trait Locus) encoding a sodium transporter. Possibly, *OsABI5* acts as an ABA-dependent negative regulator of stress tolerance in rice. Furthermore, the fertility of *OsABI5*-antisense lines was lower than in the wild type due to unsettled pollen formation. It is possible that *OsABI5* can also regulate pollen maturation (Zou et al., 2008).

In wheat, TaABI5 is closely related to HvABI5. Its expression is induced by ABA, drought and low temperature. Two-week-old seedlings of *TaABI5*-overexpression tobacco (*Nicotiana tabacum*) lines showed a better survival rate in freezing temperature (Kobayashi et al., 2008). Additionally, 7-day-old seedlings of transgenic lines were more tolerant to salt and osmotic stress and had a higher percentage of green cotyledons compared to the wild type. The opposite effect of the enhanced expression of *TaABI5* and *OsABI5* on abiotic stress tolerance may be the result of the different developmental stages of the analyzed plants. Furthermore, their function in ABA-activated signaling cannot be equivalent. The root growth of *TaABI5*-overexpresssion seedlings was ABA-hypersensitive (Kobayashi et al., 2008). Additionally, *TaABI5*-regulated stress-responsive genes, *TaDHN13, TaRAB18* and *TaRAB19*, were identified (Kobayashi et al., 2008).

*ZmABI5* in maize shows a homology to AtABI5 and HvABI5. The expression of *ZmABI5* is activated by ABA, SA (salicylic acid) salt, cold and heat stress. On the other hand, *ZmABI5* was down-regulated by drought and wounding in the leaves. However, drought and wounding stress activated *ZmABI5* in roots, while ABA and salt treatments repressed its activity. Twenty-one-day-old seedlings of tobacco *ZmABI5*-transgenic lines showed a lower tolerance to drought, salt, heat and cold compared to the wild type. Chlorophyll content, proline accumulation and the activity of antioxidant enzymes, POD (PEROXIDASE) and SOD (SUPEROXIDE DISMUTASE) were decreased in transgenic lines under drought, salt, cold and heat stress (Yan et al., 2012). After stress application, *ZmABI5*-overexpressed plants also had a higher amount of MDA (MALONDIALDEHYDE), which is an indicator of oxidative damage. Furthermore, salt and heat caused a lower induction of stress-related genes such as *CAT1, APX, ERD10A-D* (*EARLY RESPONSE TO DEHYDRATION*) and *PR5* (*PATHOGENESIS-RELATED 5*) in plants with an overexpression of *ZmABI5* compared to the wild type, while under osmotic and cold stress, the expression of these genes was up- or down-regulated. The changed expression of stress-response genes in transgenic plants indicates the complex role of *ZmABI5* in responses to adverse conditions depending on the type of the stress that is applied. It can be assumed that *ZmABI5*, like *OsABI5*, acts as a negative regulator of the abiotic stress response (Yan et al., 2012). The results presented above indicate that monocot *AtABI5* orthologs may participate in abiotic stress responses in different ways. Possibly, their action is related to the stage of plant development. Additionally, the mechanism of action of particular orthologs may not be the same in different monocot species. Furthermore, *AtABI5* orthologs can also function in different ways depending on the type of stress.

**FIGURE 3:**
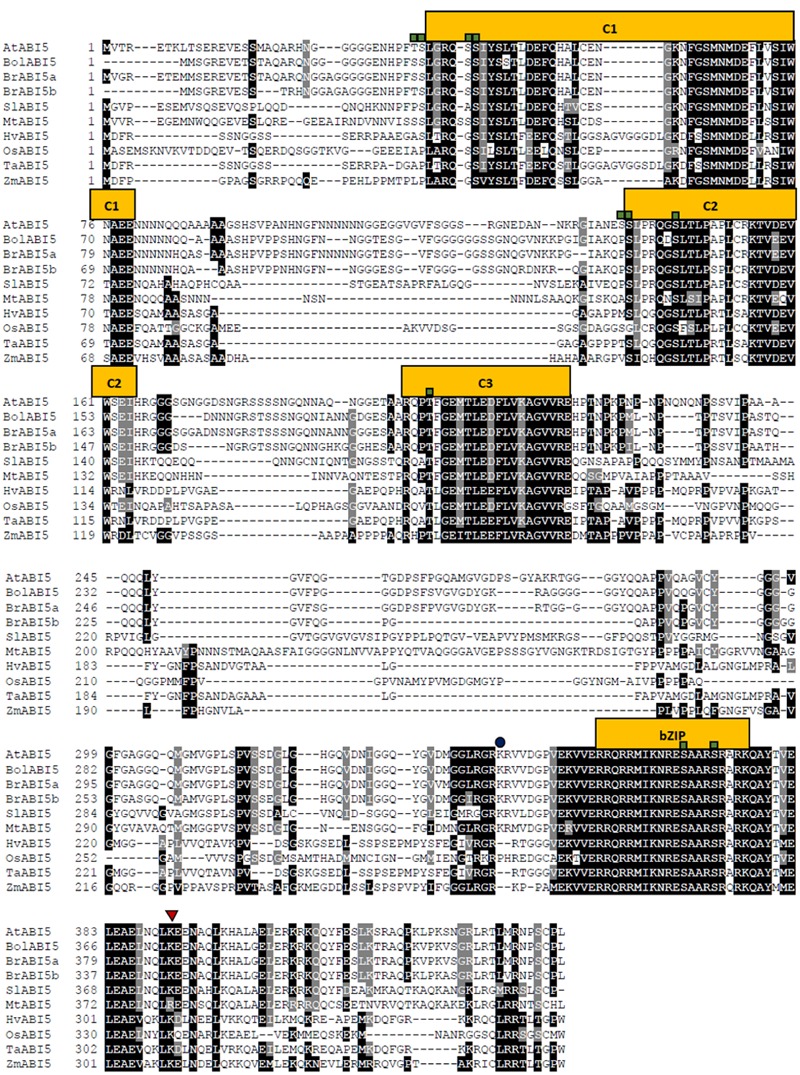
**Alignment of ABI5 proteins from 9 species including dicots and monocots orthologs.** The square, triangle and circle mark phosphorylation, sumoylation and ubiquitination sites respectively. The alignment was generated using Clustal Omega (https://www.ebi.ac.uk/Tools/msa/clustalo/) and Boxshade (http://www.ch.embnet.org/software/BOX_form.html) softwares. C1, C2, C3 – conserved regions, bZIP – basic leucine zipper domain.

**FIGURE 4:**
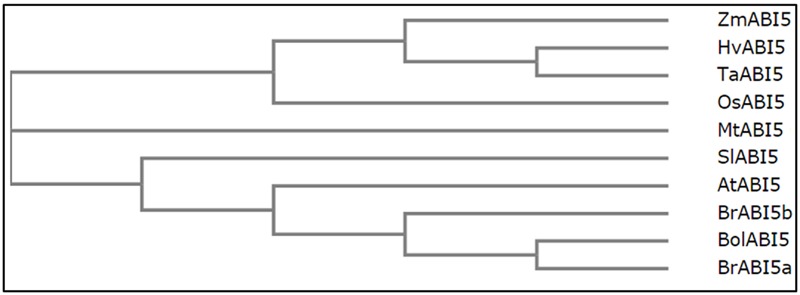
**Relationships between AtABI5 and its orthologs.** Phylogenetic tree was generated using Clustal Omega software (https://www.ebi.ac.uk/Tools/msa/clustalo/).

The multiple sequence alignment presents the level of similarity between the described dicot and monocot orthologs (**Figure 3**). Across the species, the most conserved domain in AtABI5 is a bZIP domain. Conversely, C1 conserved region seems to be weakly preserved, especially in monocots. In other species, AtABI5 phosphorylation sites are mainly maintained in conserved domains. Interestingly, the site for the ubiquitination seems to be characteristic only for the dicots. Probably, another amino acid position is ubiquitinated in monocot plants. The sumoylation site is conserved across AtABI5 homologs. The analysis of ABI5 sequences in different species shows the divergence between dicot and monocot ABI5 forms what can result in its different action in grasses. The relationship between AtABI5 dicot and monocot orthologs is also included on the phylogenetic tree (**Figure 4**). AtABI5 seems not to show higher similarity to any from described dicot orthologs. The further distance between TaABI5 and OsABI5 may reflect their contrast function under abiotic stress.

## Concluding Remarks

Plant abiotic stress responses require a complex and accurate regulation. This is achieved through the operation of many regulators that have a modulated activity depending on the water status in the environment. ABI5 seems to be an ABA signaling regulator that integrates many different signals and influences the expression of stress-responsive genes. Many processes such as seed germination, seedling growth, photosynthesis and lateral root development were shown to be regulated by ABI5. The mode of the *ABI5* gene and ABI5 protein regulation is complex and requires many transcription factors, enzymes and protein regulators. *ABI5* expression is the outcome of the action of many transcription factors such as WRKY and MYB as well as epigenetic events. Additionally, multiple protein interactions and posttranslational modifications such as phosphorylation, ubiquitination, sumoylation and *S*-nitrosylation modulate ABI5 activity at the protein level. The action of ABI5 is also related to components of auxin, CK, GA, BR and JA signaling and metabolism pathways. The complicated network of ABI5 connections often results in a positive or negative ABA signaling feedback loop. Finally, the elaborate ABI5 functions ensure a balanced and adequate, respective to the intensity of stress response to adverse conditions. Further studies that are performed using monocot *ABI5* homologs and investigations of their precise actions in the presence of abiotic stress can be very helpful in obtaining cultivars that have a better stress tolerance.

## Author Contributions

AS wrote the manuscript and prepared figures. AD-G and IS contributed to the writing of manuscript and revised it critically for important intellectual content.

## Conflict of Interest Statement

The authors declare that the research was conducted in the absence of any commercial or financial relationships that could be construed as a potential conflict of interest.
